# Suberoylanilide hydroxamic acid, an inhibitor of histone deacetylase, suppresses vasculogenic mimicry and proliferation of highly aggressive pancreatic cancer PaTu8988 cells

**DOI:** 10.1186/1471-2407-14-373

**Published:** 2014-05-27

**Authors:** Xing-dong Xu, Lan Yang, Li-yun Zheng, Yan-yan Pan, Zhi-fei Cao, Zhi-qing Zhang, Quan-sheng Zhou, Bo Yang, Cong Cao

**Affiliations:** 1 Department of General Surgery, the Third Hospital affiliated to Soochow University, Changzhou City 213003, Jiangsu, China; 2 Department of Neurology, the First Affiliated Hospital of Soochow University, Suzhou, China; 3 Cyrus Tang Hematology Center, Soochow University, Suzhou 215123, Jiangsu, China; 4 Jiangsu Key Laboratory of Translational Research and Therapy for Neuro-Psycho-Diseases and Institute of Neuroscience, Soochow University, Suzhou 215021, Jiangsu, China

**Keywords:** Pancreatic cancer, SAHA, Vasculogenic mimicry, Proliferation and apoptosis

## Abstract

**Background:**

Pancreatic cancer is one of the most aggressive human malignancies with a extremely low 5-year survival rate. Hence, the search for more effective anti-pancreatic cancer agents is urgent.

**Methods:**

PaTu8988 pancreatic cancer cells were treated with different concentrations of suberoylanilide hydroxamic acid (SAHA), cell survival, proliferation, migration and vasculogenic mimicry (VM) were analyzed. Associated signaling changes were also analyzed by RT-PCR and Western blots.

**Results:**

Here, we reported that SAHA, a histone deacetylase inhibitor (HDACi), exerted significant inhibitory efficiency against pancreatic cancer cell survival, proliferation, migration and VM. SAHA dose-dependently inhibited PaTu8988 pancreatic cancer cell growth with the IC-50 of 3.4 ± 0. 7 μM. Meanwhile, SAHA suppressed PaTu8988 cell cycle progression through inducing G2/M arrest, which was associated with cyclin-dependent kinase 1 (CDK-1)/cyclin-B1 degradation and p21/p27 upregulation. Further, SAHA induced both apoptotic and non-apoptotic death of PaTu8988 cells. Significantly, SAHA suppressed PaTu8988 cell *in vitro* migration and cell-dominant tube formation or VM, which was accompanied by semaphorin-4D (Sema-4D) and integrin-β5 down-regulation. Our evidences showed that Akt activation might be important for Sema-4D expression in PaTu8988 cells, and SAHA-induced Sema-4D down-regulation might be associated with Akt inhibition.

**Conclusions:**

This study is among the first to report the VM formation in cultured human pancreatic cancer cells. And we provided strong evidence to suggest that SAHA executes significant anti-VM efficiency in the progressive pancreatic cancer cells. Thus, SAHA could be further investigated as a promising anti-pancreatic cancer agent.

## Background

Pancreatic cancer is one of the most aggressive human malignancies, with less than 5% of patients still alive five years after diagnosis [1]. In 2012, it is estimated that a total of 43,920 patients will be diagnosed with pancreatic cancer in the United States, and 37,390 will die of this disease [2]. Pancreatic cancer is characterized by a rapid disease progression and highly invasive phenotype. Most patients are with unresectable tumor at the time of diagnosis, leaving chemotherapy and radiation as the only available treatment options [3]. For the past decades, gemcitabine has been the standard treatment for advanced pancreatic cancers, prolonging survival by 5–6 months [4]. However, a large percentage of pancreatic cancers do not respond to gemcitabine, probably due to the high level of intrinsic and acquired chemo-resistances [5].

Angiogenesis is essential for tumor growth and metastasis. Tumor-associated angiogenesis is critical for pancreatic cancer progression [6]. Several modes of vessel formation have been proposed so far: vasculogenesis, angiogenesis, intussusceptions, vascular cooption and vasculogenic mimicry (VM) [7]. VM is the process where fluid-conducting channels were formed by the highly invasive and genetically dysregulated tumor cells [8]. Tumors with high VM abilities are often highly aggressive and associated with poor prognosis [8-10]. VM has been observed in a variety of aggressive tumors including carcinomas, breast cancers, liver cancers, ovarian cancers, prostate cancers, sarcomas, gliomas and melanomas [11,12]. Pancreatic cancer represents one of the most vascularized and angiogenic solid tumors [13]. In the current study, we found that many human pancreatic cancer cells could also form tube like structure (VM) *in vitro*.

In the current study, we aimed to seek novel and more efficient treatment strategies by targeting angiogenic mimicry in pancreatic cancer cells. Suberoylanilide hydroxamic acid (SAHA) belongs to the histone deacetylases (HDAC) inhibitors (HDACi), which represent a new class of anti-cancer therapeutics. Studies have confirmed its high efficiency in inhibiting angiogenesis in pre-clinical animal models and early phase clinical trials [14,15]. SAHA inhibits the *in vitro* and *in vivo* growth of transformed human cancer cells, including prostate, bladder and ovarian tumor cells [15,16]. SAHA has been tested in phase I and phase II clinical trials for the treatment of various malignancies, and has demonstrated significant anti-cancer efficiency at well-tolerated doses [15,16]. Meanwhile, studies have shown that SAHA exhibits profound inhibitory effects against human pancreatic cancer cells [17]. However, the potential effect of SAHA on VM and proliferation of highly metastasis pancreatic cancer cells is not fully studied. Further, the underlying mechanisms remain inconclusive. In this study, we found that SAHA inhibits *in vitro* proliferation, migration and VM in a highly aggressive human pancreatic cancer cells (PaTu8988).

## Methods

### Chemical and reagents

SAHA (Purity ≥99%) was purchased from Selleck Chemicals (Houston, TX). Matrigel and the anti-Semaphorin-4D (Sema-4D) antibody were obtained from BD Biosciences (San Jose, CA). Trypan blue was purchased from Beyotime Biotechnology (Shanghai, China). Annexin V-FITC apoptosis detection kit was purchased from Biotech Co., Ltd (Nanjing, China). RNase-free DNase I was from Qiagen (Hilder, Germany). RevertAid™ First Strand cDNA Synthesis Kit was purchased from Fermentas Life Sciences (Chicago, IL). Taq™ DNA Polymerase was from TaKaRa Biotechnology Co., Ltd (Dalian, China). Propidium iodide (PI), monoclonal antibody against β-actin and gelatin were obtained from Sigma (St. Louis, Mo). The anti-cyclin-D1 antibody was obtained from ABGENT (Suzhou, China). Anti-epidermal growth factor receptor (EGFR) and platelet-derived growth factor receptor (PDGFR) antibodies were purchased from Santa Cruz Biotech (Santa Cruz, CA). Akt, p-Akt (Ser 473), p70S6 kinase (S6K1), p-S6K1 (Thr 389), S6, p-S6 (Ser 235/236), mTOR, p-mTOR (Thr 289), Ulk1, p-Gsk-3β, Ulk1, Erk1/2 and p-Erk1/2 antibodies were purchased from Cell Signaling Tech (Beverly, MA). Primers were synthesized by GENEWIZ, Inc. (Suzhou, China).

### Cell culture

As previously described [18], human pancreatic cancer cell lines PaTu8988, Bxpc-3, Aspc-1, CFPAC-1, PaTu8988, SW1990, Panc-1 as well as normal hypertrophic scar fibroblasts (HSF) were obtained from Chinese Academy of Sciences Cell Bank (Shanghai, China). Cells were cultured in RPMI (HyClone, Shanghai, China) with 10% heat-inactivated fetal bovine serum (FBS), with 100 U/ml of penicillin G and 100 μg/ml of streptomycin in a 5% CO_2_ incubator at 37°C. Fresh peripheral blood mononuclear cells (PBMNCs) from three healthy adults were collected and separated by Ficoll-Hipaque density sedimentation as previously reported [18], the cells were then cultured in RPMI 1640 medium supplemented with 10% heat-inactivated FBS, 100 U/ml penicillin G and 100 μg/mL streptomycin. The study was approved by the institutional review board of the Third Hospital affiliated to Soochow University and all other authors’ institutions, and written informed consent was obtained from all three human participants. All clinical investigations were conducted according to the principles expressed in the Declaration of Helsinki.

### Cell growth assay

Pancreatic cancer PaTu8988 cell growth was assessed using the trypan blue exclusion test. Cells were seeded in 6-well plates for 24 h, various concentration of SAHA was added, cells were further cultured for additional 48 h. Afterwards, cells were harvested and stained with trypan blue. The unstained ("survival") cells were counted in a Neubauer chamber, and the number was expressed as the percentage change of control group. The IC-50, defined as the drug concentration at which cell growth was inhibited by 50%, was assessed by SPSS 16.0 software. All experiments were repeated at least three times.

### Colony formation assay

PaTu8988 cells treated with SAHA for 48 h were harvest, a total of 1 × 10^3^ cells per well suspended in 150 μL of Mix agar with 1.5 mL DMEM/10% FBS were plated in 30 mm plates overlying a 1% agar-DMEM/10% FBS(1:1) bottom layer. After 3 weeks, colonies were photographed at 4×. The remaining survival large colonies (>100 μM in diameter) were manually counted.

### Cell cycle assay

PaTu8988 cells were grown in T75 flasks and treated with indicated dosage of SAHA for 48 h. After the treatment, the cells were fixed with 70% ethanol overnight at 4°C, washed with PBS, re-suspended in 500 μL PBS with 100 μg/mL RNase and incubated for 30 min at 37°C. After that, 2.5 μL of PI solution (10 mg/mL) was added. The DNA contents of PI-stained cells were analyzed using a flow cytometry (Becton Dickinson FACS Calibur).

### Cell apoptosis assay

PaTu8988 cell apoptosis was detected by the Annexin V Apoptosis Detection Kit (Beyotime, Shanghai, China) according to the manufacturer’s protocol. Briefly, one million cells with indicated treatments were stained with FITC-Annexin V and PI (Beyotime, Shanghai, China). Both early (annexin V^+^/PI^-^) and late (annexin V^+^/PI^+^) apoptotic cells were sorted by fluorescence-activated cell sorting (FACS) (Becton Dickinson FACS Calibur).

### Cell morphologic analysis

A total of 4 × 10^4^ PaTu8988 cells were seeded on glass cover slips in the six well plate and treated with the indicated concentration of SAHA for 48 h. Cells were fixed and stained with Wright-Giemsa stain. The slides were photographed using oil microscopy (×1000 magnification).

### In vitro tube formation assay or vasculogenic mimicry (VM) assay

The tumor cell formation of capillary structure *in vitro* was tested as we previously described [19,20].

### Cellular immuno-fluorescence staining

PaTu8988 cells were seeded on glass cover slips in six well plates and treated with described dosage of SAHA for 48 h. Cells on the cover slip were then fixed with 4% paraformaldehyde for 10 min at room temperature without permeabilization. Slides were washed three times with phosphate buffered saline (PBS), blocked with 5% bovine serum albumin (BSA) for 1 h at 37°C, followed by incubation with the primary antibody overnight at 4°C, and the secondary antibody for 1 h at room temperature. The slides were photographed using OLYMPUS FSX-100 microscope.

### MTT cell viability assay

The cell viability was measured by the 3-[4,5-dimethylthylthiazol-2-yl]-2,5 diphenyltetrazolium bromide (MTT) method, as described before [21]. Briefly, the PaTu8988 cells were collected and seeded in 96-well plate at a density of 2 × 10^5^ cells/cm^2^. Different seeding densities were optimized at the beginning of the experiments. After treatment, 20 μl of MTT tetrazolium (Sigma, St. Louis, MO) salt dissolved in PBS at a concentration of 5 mg/mL was added to each well and incubated in a CO_2_ incubator for additional 2 hrs. Finally, the medium was aspirated very carefully and 150 μl/well of DMSO (Sigma, St. Louis, MO) was added to dissolve formazan crystals. The absorbance of each well was obtained using a plate reader at a test wavelength of 490 nm with a reference wavelength of 630 nm. The value of treatment group was always normalized to that of control group.

### "Scratch" assay

As described [22], twelve-well plates were pre-coated with poly-lysine (30 μg/ml), followed by further BSA blocking. A sufficient number of PaTu8988 cells were plated, so that they became confluent in the wells right after attachment (~1–2 h). Same area of each well is then displaced by scratching a same straight line through the layer with a needle. Floating cells were washed away by warm PBS. Cells were further incubated with the indicated concentration of SAHA for 24 h, and stained with Wright-Giemsa to see migration "gap". Mitomycin C (10 μg/ml) was always included in the culture media to prevent cell proliferation.

### PCR analysis

Total RNA was extracted from PaTu8988 cells and treated with RNase-free DNase I. The quality of RNA was test by DU-800 Nucleic Acid/Protein Analyzer (Beckman, U.S.A). The cDNA was generated by reverse transcription using RevertAidTM First Strand cDNA Synthesis Kit and oligo (dT) in a 20 μL reaction containing 5 μg of total RNA. Next, PCR was performed in each 25 μL PCR reaction containing 0.5 μL diluted cDNA, TaKaRa rTaq DNA Polymerase and indicated primers. The PCR reaction contained an initial denaturation at 94°C for 3 min, followed each PCR cycle by de-naturation at 94°C for 30 seconds, annealing at 55–68°C for 30 seconds, and extension at 72°C for 1 min for a total of 22–36 cycles, depending on the primer length and the molecular weights of target genes. PCR products were analyzed by 1.5% agarose gel. Primers used in this study were summarized in Table 1.

**Table 1:** **Primer sequences for semi**-**quantitative RT**-**PCR**

**Gene name (mRNA ID)**	**Primer name**	**Primers sequences (5’-3’)**	**PCR product size (bp)**
β-actin	Forw	AAGAGCTACGAGCTGCCTGACG	420
(NM_001101.3)	Rev	CGCCTAGAAGCATTTGCGGTGG	
Cyclin D1	Forw	CATCTCTGTACTTTGCTTGCTCAT	499
(NM_053056.2)	Rev	CGCTATTTCCTACACCTATTGGAC	
Cyclin B1	Forw	TCAACATGGCAGGCGCAAAGC	224
(NM_031966.3)	Rev	TGGCACTGGCACCAGCATAGGT	
CDK1	Forw	TGCTGGGGTCAGCTCGTTACTCA	232
(NM_001786.4)	Rev	TGGGATGCTAGGCTTCCTGGTT	
p53	Forw	GGGAGTAGGACATACCAGCTTAGAT	452
(NM_000546.4)	Rev	TTAGGTACTAAGGTTCACCAAGAGG	
p21	Forw	CTGCCTTAGTCTCAGTTTGTGTGT	412
(NM_000389.3)	Rev	CAAAGTGCCATCTGTTTACTTCTC	
CDK4	Forw	CTTGATCTGAGAATGGCTACCTCT	409
(NM_000075.2)	Rev	CATGAAGGAAATCTAGGCCTCTTA	
MAGEF1	Forw	GGGTATCCGAAGAGGCTTATTATGG	749
(NM_022149.4)	Rev	AAGCAAATGAAGGTACATGCCAGTC	
FGFR1	Forw	GAGGAGAAAGAAACAGATAACACCA	427
(NM_023108.2)	Rev	TGTACACCTTACACATGAACTCCAC	
HIF1A	Forw	GTAAGAAGGCAGTAACCTTTCATCA	502
(NM_001530.3)	Rev	AGGGTAGGCAGAACATTTAGGTTTA	
RUNX1	Forw	AGATTTAATGACCTCAGGTTTGTCG	328
(NM_001754.4)	Rev	GACTCTGAGGCTGAGGGTTAAAG	
Sema4D	Forw	TGTCTGTGGAGTATGAGTTTGTGTT	552
(NM_006378.3)	Rev	GGGTGTAGTTCACATCTTTCTTGAT	
VEGFA	Forw	AGAAGAGACACATTGTTGGAAGAAG	439
(NM_003376.4)	Rev	CGGTACAAATAAGAGAGCAAGAGAG	
MAGED1	Forw	TCGGTCTCCTCTTGGTGATTCTGG	200
(NM_006986.3)	Rev	GTTGCTGTTGGGCACTCGTCTGT	
Intergrin β5	Forw	TGCCATGCAGGTTACATCGG	353
(NM_002213)	Rev	ATCATGACGCAGTCCTTGGC	
WIPF2	Forw	CTCCTTTACTCAGACATAGAGCATCA	484
(NM_133264.4)	Rev	AGACCACCCAATCTTCCACGAA	
ZFP106	Forw	CCCACCTCTCACCCCTATTTCCTA	280
(NM_022473.1)	Rev	AACACCTCACCTACCCGTTTCCTG	

Note: Forw: the forward primer; Rev: the reverse primer; CDK1: cyclin-dependent kinase 1; CDK4: cyclin-dependent kinase 4; FGFR1: fibroblast growth factor receptor 1; HIF1A: hypoxia inducible factor 1, alpha subunit; MAGED1: melanoma antigen family D1; FGFR1:fibroblast growth factor receptor 1; VEGFA: vascular endothelial growth factor A; Sema4D:Sema domain, immunoglobulin domain (Ig), transmembrane domain and short cytoplasmic domain, 4D; WIPF2: WAS/WASL interacting protein family, member 2; MAGEF1: melanoma antigen family F1; MAGED1: melanoma antigen family D1; ZFP106: zinc finger protein 106 homolog; RUNX1: runt-related transcription factor 1.

### Western blot analysis

As described before [21], aliquots of 30–40 μg of protein from each sample (treated as indicated in the legends) was separated by 10% SDS–polyacrylamide gel electrophoresis (SDS-PAGE) and transferred onto a polyvinylidene difluoride (PVDF) membrane (Millipore, Bedford, MA). After blocking with 10% instant nonfat dry milk for 1 h, membranes were incubated with the specific antibody overnight at 4°C, followed by incubation with corresponding secondary antibody (HRP-conjugated anti-rabbit or anti-mouse IgG at the appropriate dilutions) for 30 min to 1 h at room temperature. Antibody binding was detected with the enhanced chemiluminescence (ECL) detection system (Amersham Biosciences, Piscataway, NJ). The intensity of interested band was quantified using ImageJ software, and the value was normalized to corresponding loading controls.

### Statistic analysis

The data shown in this study represented the mean ± S.E. Differences between the groups were assessed by one-way ANOVA using SPSS 16.0 software. The significance of differences was indicated as *P < 0.05 and **P < 0.01.

## Results

### SAHA inhibits the proliferation of PaTu8988 pancreatic cancer cells

**Figure 1:**
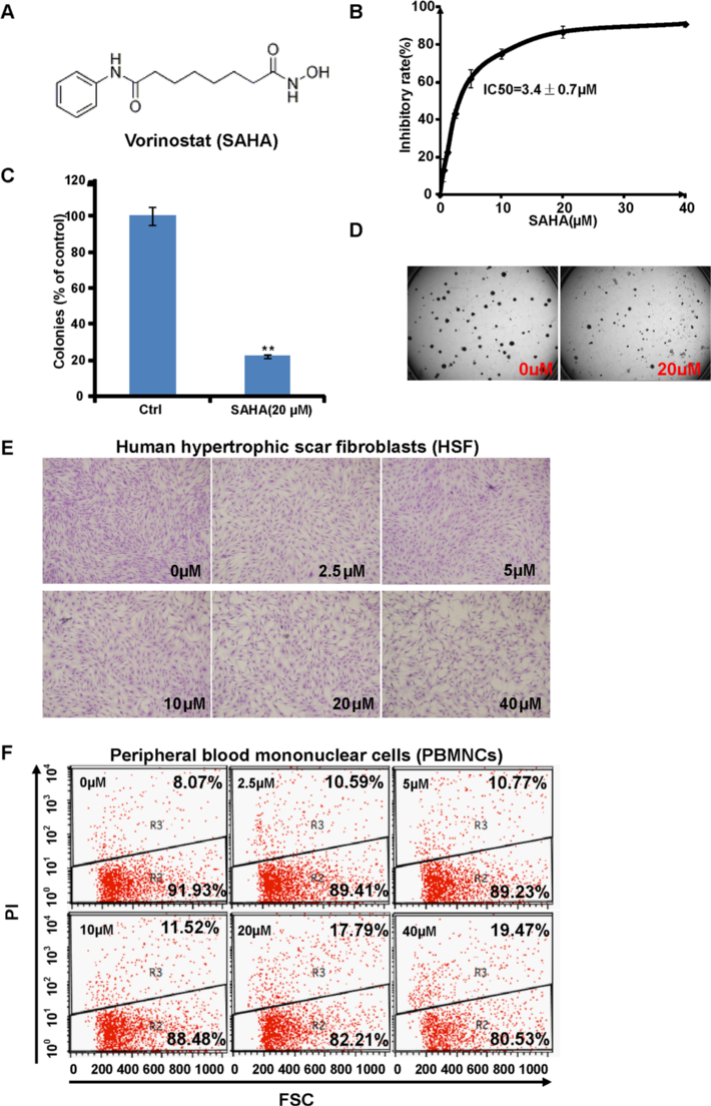
***SAHA inhibits the proliferation of PaTu8988 pancreatic cancer cells*.** Chemical structure of SAHA is shown in **(A)**. Human pancreatic cancer PaTu8988 cells were incubated with SAHA at indicated dosage for 48 h, and cell growth was measured by trypan blue exclusion test. The IC-50 was calculated by SPSS 16.0 software **(B)**. PaTu8988 cells were treated with SAHA (20 μM). The colony formation assay was performed, and the number of colonies was manually counted **(C and D)**. Human hypertrophic scar fibroblasts (HSF) were treated with various concentration of SAHA for 48 h, followed by Wright-Giemsa staining and photograph by OLYMPUS FSX-100 microscope **(E)**. Peripheral blood mononuclear cells (PBMNCs) were treated with various concentration of SAHA for 48 h, cell death was detected by PI staining through FACS sorting **(F)**. Experiments in this figure were repeated three times, and similar results were obtained. The data in this figure was expressed as mean ± S.E. **P < 0.01 vs. Ctrl **(C)**. Magnification: 1:100 **(E)**.

Figure 1A showed the chemical structure of SAHA. Considering that uncontrolled proliferation and robust angiogenesis (i.e. VM) contribute to the growth and metastasis of pancreatic cancers, we first investigated the potential role of SAHA on the pancreatic cancer cell proliferation. As shown in Figure 1B, SAHA dose-dependently inhibited PaTu8988 cell proliferation with the IC-50 of 3.4 ± 0.7 μM. However, it had almost no effect on the proliferation of HSF (hypertrophic scar fibroblasts) (Figure 1E) and normal PBMNCs (peripheral blood mononuclear cells) (Figure 1F) at the dose up to 40 μM. These results suggested that SAHA has selective inhibitory efficiency against pancreatic cancer cells, but not normal mononuclear cells or HSF cells. To further explore the inhibitory ability of SAHA on PaTu8988 cell proliferation under more stringent conditions, the colonial survival assay was performed. The results showed that the number of remaining survival colonies in SAHA-treated group was significantly lower than that of control group (Figure 1C and D). Hence, these results demonstrated that SAHA effectively inhibits PaTu8988 cell *in vitro* proliferation.

### SAHA affects cell cycle progression of PaTu8988 cells

**Figure 2:**
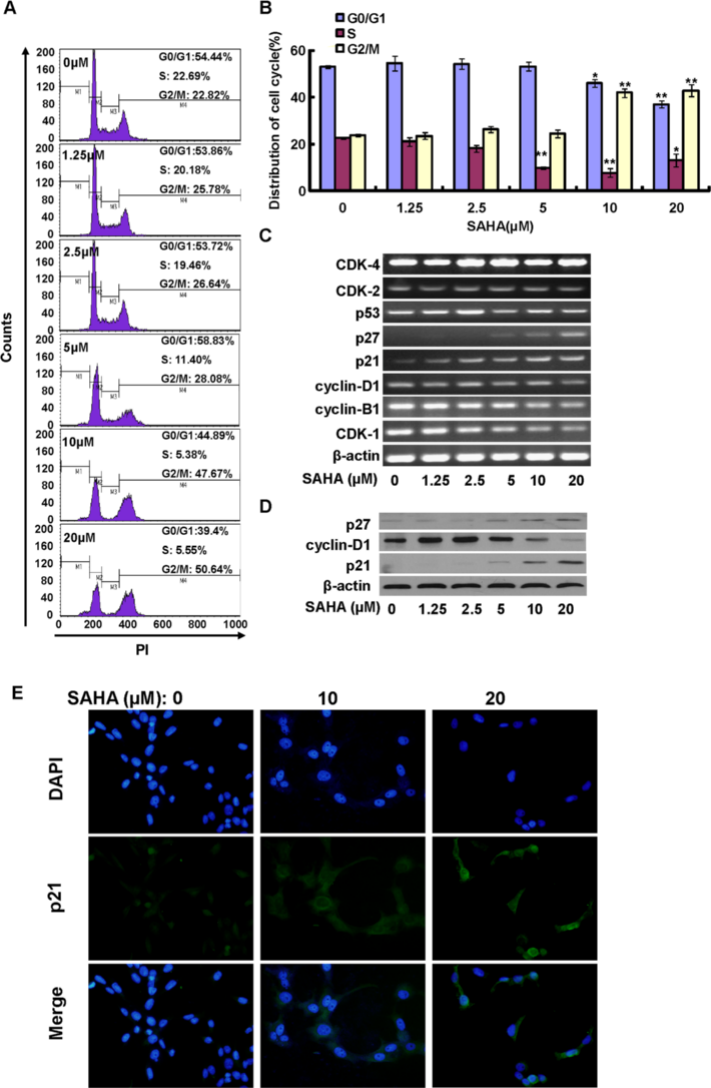
***SAHA affects cell cycle progression of PaTu8988 cells*.** PaTu8988 cells were incubated with SAHA at indicated dosage for 48 h, DNA content of PI-stained cells was analyzed by flow cytometry **(A)**, and cell cycle distribution was analyzed **(B)**. The mRNAs of cell cycle regulatory genes were detected by semi-quantitative RT-PCR assay **(C)**. The protein expressions of cyclin-D1, p21, p27 and β-actin were also tested by western blots **(D)**, and the expression and sub-cellular location of p21 were also measured by immuno-fluorescence **(E)**. Experiments in this figure were repeated three times, and similar results were obtained. The data in this figure was expressed as mean ± S.E. *P < 0.05 vs. Ctrl, **P < 0.01 vs. Ctrl **(B)**. Magnification: 1:400 **(E)**.

Next, we analyzed the cell cycle distribution in SAHA-treated PaTu8988 cells. As shown in Figure 2A and B, a large population of SAHA (>5-10 μM)-treated PaTu8988 cells were arrested in G2/M phase. Meanwhile, RT-PCR results showed that the mRNA expressions of cyclin-dependent kinase 1 (CDK-1), cyclin-D1 and cyclin-B1 were down-regulated after SAHA treatment, while the p21 and p27 mRNAs were markedly increased (Figure 2C). The CDK-2, CDK-4 and p53 mRNAs were not affected by SAHA (Figure 2C). Further, western blot results in Figure 2D confirmed that the protein level of cyclin-D1 was markedly decreased after SAHA (10 or 20 μM) treatment, while p21 and p27 protein expressions were significantly upregulated. Immuno-fluorescence results in Figure 2E further confirmed p21 upregulation and nuclear translocation after SAHA stimulation in PaTu8988 cells. These results suggested that SAHA suppresses cell cycle progression by inducing G2/M arrest in PaTu8988 cells; such effect of SAHA is associated with perturbation of cell cycle-associated proteins.

### SAHA induces both apoptotic and non-apoptotic death of PaTu8988 cells

**Figure 3:**
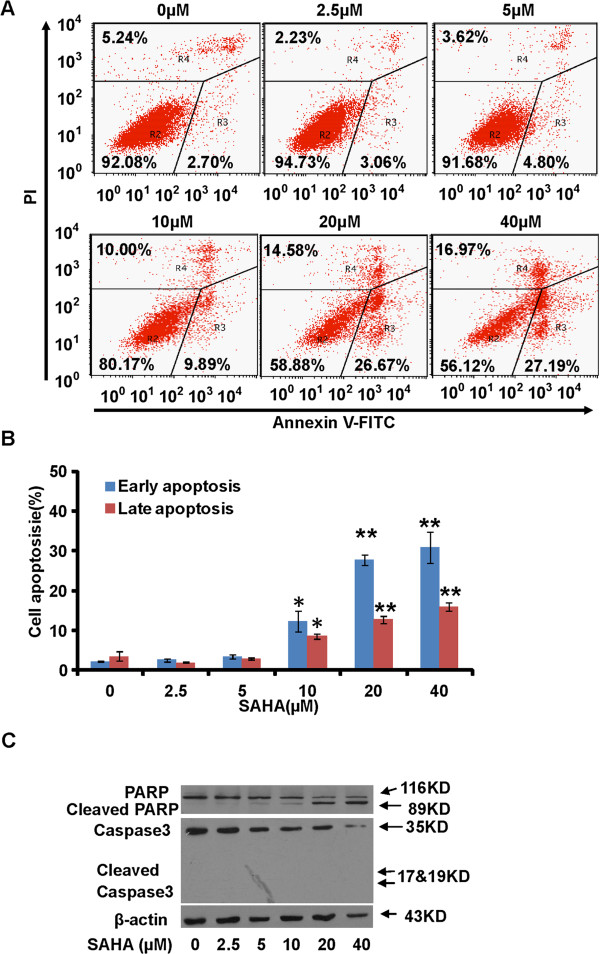
***SAHA induces both apoptotic and non*- *apoptotic death of PaTu8988 cells*.** PaTu8988 cells were incubated with SAHA at indicated dosage for 48 h, FITC-annexin V and PI stained cells were sorted by flow cytometry **(A)**. The distribution of cell apoptosis was analyzed **(B)**. The protein expressions of PARP, cleaved-PARP, caspase-3, cleaved-caspase-3 and β-actin were detected by western blots **(C)**. Experiments in this figure were repeated three times, and similar results were obtained. The data in this figure was expressed as mean ± S.E. *P < 0.05 vs. Ctrl, **P < 0.01 vs. Ctrl **(B)**.

Next, we examined whether the inhibitory effect of SAHA on PaTu8988 cell proliferation was due to cell apoptosis. As shown in Figure 3A and B, the population of apoptotic (Annexin V positive) PaTu8988 cells increased significantly after high dose SAHA (>10 μM) treatment. Meanwhile apoptosis-associated proteins were also changed. Poly (ADP-ribose) polymerase (PARP) and caspase-3 were down-regulated after SAHA treatment, while cleaved-PARP was up-regulated (Figure 3C). We failed to see an increase of cleaved-caspase-3 in SAHA-treated PaTu8988 cells (Figure 3C). Interestingly, we also noticed a small population of non-apoptotic "dead" PaTu8988 cells (Annexin V negative and PI positive) after SAHA treatment. Together, these results suggested that both apoptotic and non-apoptotic cell death might contribute to SAHA-induced anti-proliferation effect in PaTu8988 cells.

### SAHA induces differentiation and inhibits migration of PaTu8988 cells

**Figure 4:**
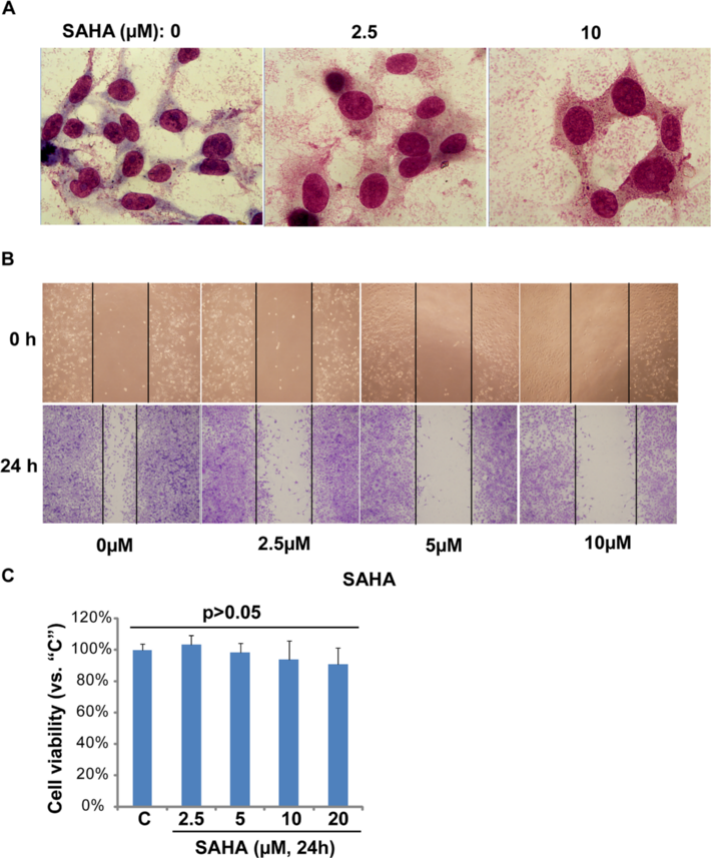
***SAHA induces differentiation and inhibits migration of PaTu8988 cells*. (A)** PaTu8988 cells were seeded on glass cover slips in six-well plate and treated with SAHA for 48 h. Cells were stained with Wright-Giemsa stain and photographed by oil microscopy for 1000×. **(B)** PaTu8988 cells were incubated with SAHA at indicated dosage for 24 h, the *in vitro* cell migration was analyzed by "scratch" assay as described, cell viability was analyzed by MTT assay **(C)**. Experiments in this figure were repeated three times, and similar results were obtained. Magnification: 1:200 **(B)**.

We also examined the potential effect of SAHA on the morphology change of PaTu8988 cells. The PaTu8988 cells were incubated with SAHA for 48 h. Afterwards, cells were stained with Wright-Giemsa to see their morphology. As shown in Figure 4A, control cells were small and had little hyper-chromatism in cytoplasm, indicating an undifferentiated shape. While the SAHA-treated cells were bigger, and were with full of light cytoplasm and cytoplasm projections: a typical differentiated shape. These results suggested that SAHA might induce PaTu8988 cell differentiation. We also tested the effect of SAHA on cell migration through *in vitro* "scratch" assay; results in Figure 4B demonstrated that SAHA dose-dependently suppressed the "gap" closing, indicating its inhibitory efficiency against PaTu8988 cell *in vitro* migration. The inhibitory effects of SAHA on cell migration were not secondary to decreased viability, as no significant cell viability decrease was observed after indicated SAHA treatment for 24 h (Figure 4C).

### SAHA suppresses PaTu8988 cell vasculogenic mimicry (VM)

**Figure 5:**
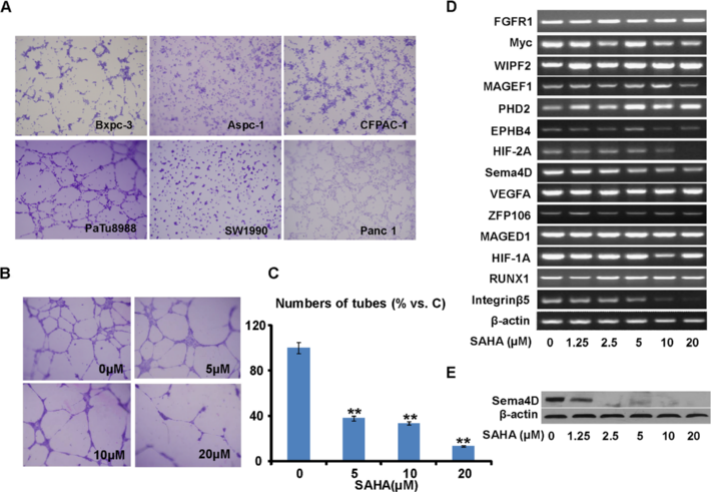
***SAHA suppresses PaTu8988 cell vasculogenic mimicry* (*VM*).** The six human pancreatic cancer cells including Bxpc-3, Aspc-1, CFPAC-1, PaTu8988, SW1990 and Panc-1 were harvested and suspended in RPMI-1640 plus 10% FBS at a density of 2 × 10^5^/mL. A volume of 150 μL/well of Matrigel matrix was transferred to a 48-well plate at 37°C for 30 min. Then the cells were transferred to each well. The cells were further incubated at 37°C, 5% CO_2_ for 4–6 h, the formed tubes were stained with Wright-Giemsa and photographed by OLYMPUS FSX-100 microscope **(A)**. PaTu8988 cells were treated with the indicated concentration of SAHA for 24 h, and subjected to a tube formation assay as described **(B)**. The capillary-like structures were imaged and analyzed for quantification **(C)**. The mRNAs of vasculogenic and angiogenic genes were determined by semi-quantitative RT-PCR assay **(D)**, and the expression of Sema-4D was detected by western-blot **(E)**. Experiments in this figure were repeated three times, and similar results were obtained. The data in this figure was expressed as mean ± S.E. **P < 0.01 vs. Ctrl **(C)**. Magnification: 1:200 **(A and B)**.

Results above have shown that SAHA inhibits PaTu8988 cell *in vitro* migration. VM is the formation of fluid-conducting channels by highly invasive and genetically dysregulated tumor cells [9]. Through *in vitro* tube formation assay, we observed the VM formation in multiple human pancreatic cancer cells (Bxpc-3, PaTu8988, Panc-1 and CFPAC-1) (Figure 5A). To examine whether SAHA have anti-VM ability, the PaTu8988 cells, pretreated with or without SAHA, were seeded onto a Matrigel layer and the capillary tube formation ability was monitored and photographed. As shown in Figure 5B-C, the PaTu8988 cells again formed a good tube like structure, which was inhibited by SAHA. Note that 20 μM of SAHA almost completely disrupted VM formation. VM-associated genes were also tested in control and SAHA-treated PaTu8988 cells. As shown in Figure 5D, Sema-4D and integrin-β5 mRNAs were significantly down-regulated by SAHA (10 and 20 μM), and the HIF-2A mRNA expression was also suppressed by SAHA (20 μM). Interestingly, other tumor VM and angiogenic genes including RUNX1, HIF (Hypoxia-inducible factor)-1A, integrin-α5 and VEGF (vascular endothelial growth factor)-A were not affected. Further, western blot results (Figure 5E) confirmed that Sema-4D protein was down-regulated by SAHA in PaTu8988 cells. Hence, these results suggested that SAHA inhibited PaTu8988 cell *in*-*vitro* VM, which was associated with Sema-4D and integrin-β5 down-regulation.

### Akt is important for Sema-4D expression in PaTu8988 cells, inhibited by SAHA

**Figure 6:**
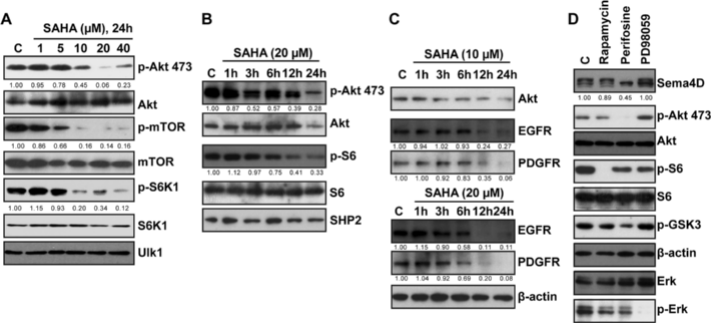
***Akt is important for Sema*-*4D expression in PaTu8988 cells***, ***inhibited by SAHA*.** PaTu8988 cells were stimulated with indicated concentration of SAHA, cells were further cultured for indicated time, expressions of p-Akt (Ser 473), Akt, p-mTOR (Ser 2448), mTOR, p-S6K1 (Thr 389), S6K1, p-S6 (Ser 235/236), S6, SHP-2, EGFR, PDGFRβ and β-actin were detected by western blots **(A-C)**. PaTu8988 cells were treated with Akt inhibitor perifosine (10 μM), mTORC1 inhibitor rapamycin (100 nM) or Erk/MAPK inhibitor PD98059 (10 μM), cells were further cultured for 24 h, expressions of p-Akt (Ser 473), Akt, p-S6 (Ser 235/236), S6, p-Gsk3β, β-actin, Erk1/2, p-Erk1/2 and Sema-4D were tested by western blots. Akt/mTOR/S6K1 phosphorylation in **(A)**, Akt/S6 phosphorylation in **(B)**, EGFR/PDGFRβ expression in **(C)**, and Sema-4D expression in **(D)** were quantified after normalization to corresponding loading controls, and the number was presented as fold change vs. untreated **("C")** control group. Experiments in this figure were repeated three times, and similar results were obtained.

Since previous studies have confirmed that Akt and its downstream mTORC1 is important for both survival and migration of pancreatic cancer cells [23-29], we thus wanted to know whether SAHA could affect activation of Akt-mTORC1 in PaTu8988 pancreatic cancer cells. Also, it has been suggested that Akt signaling is linked with cancer cell VM [30,31], we tested whether this signaling pathway was important for Sema-4D expression. As shown in Figure 6A and B, SAHA (10–40 μM) significantly inhibited activation of Akt. Meanwhile, mTORC1 activation, indicated by p-mTOR, p-S6K1 and p-S6, was also suppressed by SAHA (Figure 6A and B). Expression of Ulk1, an indicator of autophagy activation, was not affected by SAHA treatment (Figure 6A). We proposed that growth factor receptors’ degradation might be responsible for Akt-mTORC1 inhibition by SAHA, since SAHA administration down-regulated epidermal growth factor receptor (EGFR) and platelet-derived growth factor receptor β (PDGFRβ) expression (Figure 6C). Interestingly, as shown in Figure 6D, the Akt inhibitor perifosine, but not the mTORC1 inhibitor rapamycin, inhibited Sema-4D expression in PaTu8988 cells, indicating that Akt rather than mTORC1 is important for Sema-4D expression. Even more intriguingly, although perifosine blocked Akt activation, it only inhibited, but not blocked S6 phosphorylation. These results suggested that other upstream signals beside Akt might also be responsible for mTORC1 or S6 activation in this particular cell line, and that SAHA’s inhibitory ability on mTORC1 activation might not solely depend on Akt inhibition.

## Discussion

Gemcitabine is the only standard chemotherapy for pancreatic cancer patients. However, the median survival with gemcitabine treatment was still a dismal 5.65 months with 1-year survival rate of 18% [32]. In the current study, we used PaTu8988 pancreatic cancer cells as a cell model to investigate anti-cancer activity of SAHA. Our results demonstrated that SAHA exerted profound inhibitory efficiency against PaTu8988 cells. SAHA dramatically inhibited PaTu8988 cell survival, proliferation, migration, and more importantly tuber formation or VM. This study is among the first to report the VM formation in human pancreatic cancer cells. Further, we provided strong evidence to suggest that SAHA executed a significant anti-VM effect in human pancreatic cancer cells. Meanwhile, SAHA also promoted cancer cell cycle arrest and cell death (both apoptotic and non-apoptotic). Thus, SAHA could be further investigated as a promising anti-pancreatic cancer agent.

SAHA induces PaTu8988 cell cycle arrest at G2/M phase probably via down-regulating cyclin B1. Previous studies have shown that cyclin B1 degradation is actively involved in G2/M arrest [33]. And constitutive activation of cyclin B1 overrides p53-mediated G2/M arrest [32]. In our study, we found that SAHA induced expressions of CDK inhibitors p21 and p27, which are known to affect G2/M cycle progression [34]. Here we observed a significant cell apoptosis after high dose of SAHA treatment, the mechanism of SAHA-induced apoptosis may be associated with PARP and caspase-3 degradation, as suggested by other studies [34,35]. Intriguingly, SAHA also induced non-apoptotic cell death in PaTu8988 cells. This result is not surprising, as recent studies have observed non-apoptotic death, in particular autophagic cell death induced by SAHA [36,37].

Tumor vasculogenic mimicry (VM), which is characterized by the tumor cell-lined vessels, was first found from metastatic melanoma by Hendrix MJ group in 1999 [34]. Hence, VM has been targeted for anti-cancer therapy. Here we first reported that multiple pancreatic cancer cell lines formed a good tube like structure in Matrigel *in vitro*. Significantly, SAHA greatly inhibited PaTu8988 cell-mediated VM *in vitro*, such an effect was associated with down-regulating Sema-4D and integrin-β5, two key VM associated proteins.

Here we observed a significant down-regulation of Sema-4D by SAHA in PaTu8988 cells. Sema-4D expression is seen in a wide range of human tumors including prostate, colon, breast, oral, head and neck carcinomas [37]. Sema-4D is a cell surface membrane protein that is shed from tumor cells and promotes endothelial cell proliferation, migration, angiogenesis, and tumor invasive growth through its action on its cognate endothelial receptor, plexin B1 [35]. In the absence of Sema-4D, tumor growth and tumor angiogenesis *in vivo* are greatly impaired [36]. Researchers have demonstrated that Sema-4D can potentiate the invasiveness of pancreatic cancer cells. In the present study, we found that SAHA downregulated Sema-4D expression in PaTu8988 cells, which may be one the mechanism responsible for VM disruption. To our knowledge, this is the first report showing SAHA affects Sema-4D expression and cancer cell VM.

Integrin β5 is another potent angiogenic gene whose expression in PaTu8988 cells was also suppressed by SAHA. Integrins are a family of non-covalently associated het-erodimeric cell surface receptors composed of a α and β subunit that mediate cell-ECM and cell-cell adhesions [38]. It is reported that mice lack of integrin β3 and β5 showed less tumorigenesis [39]. We found that PaTu8988 cells treated with SAHA showed inhibited expression of integrin β5, another mechanism to explain SAHA’s anti-angiogenic potential.

Pancreatic cancers are among the most intrinsically resistant tumors to almost all classes of cytotoxic drugs [38]. The extremely high level of drug resistance was associated with dysregulation of multiple signaling pathways [39,40]. One key signaling pathway that is frequently over-activated in pancreatic cancer is Akt/mTOR signaling cascade [39,40], which is responsible for cancer cell survival, proliferation, apoptosis-resistance, migration and metastasis [39,40]. The fact that SAHA significantly inhibited Akt and S6 activation in PaTu8988 cells might explain its inhibitory efficiency against this cell line. As a matter of fact, our data showed that perifosine, the Akt inhibitor, significantly inhibited PaTu8988 cell proliferation, migration and survival (Data not shown).

Importantly, recent studies have indicated that Akt signaling is also important for cancer cell vasculogenic mimicry [30,31]. In PaTu8988 cells, both Akt inhibitor perifosine [41] and SAHA inhibited Sema-4D expression. Thus SAHA-exerted inhibitory effect against VM could also be associated Akt inhibition. More direct evidence is, however, needed to further support this hypothesis. In many cancer cells, over-expression or over-activation of growth factor receptors (i.e. EGFR, PDGFR) causes Akt hyper-activation [42]. Various inhibitors have been developed to target cell surface receptors or Akt (i.e. perifosine) for clinical use against cancers [42-45]. We found that SAHA significantly down-regulated EGFR and PDGFR expressions in PaTu8988 cells, which might be responsible for Akt inhibition. Once again, more direct evidence is still needed.

## Conclusions

In summary, the above data demonstrated that SAHA possesses its anti-pancreatic cancer ability by inducing cell cycle arrest and cell apoptosis as well as suppressing tumor *in vitro* cell migration and VM. Akt inhibition might be associated with SAHA’s inhibitory efficiency. Thus SAHA may be a potential anti-VM candidate for anti-pancreatic cancer therapy.

## Abbreviations

SAHA: Suberoylanilide hydroxamic acid; VM: Vasculogenic mimicry; Sema-4D: Semaphorin-4D.

## Competing interests

The authors have no conflict of interests. There were no financial competing interests.

## Authors’ contributions

XX, LZ, LY, YP, ZC, ZZ and QZ carried out the experiments. XX, LY, LZ, CC and BY participated in the design of the study and performed the statistical analysis. XX, LY, CC and BY conceived of the study, and participated in its design and coordination and helped to draft the manuscript. All authors read and approved the final manuscript.

## Pre-publication history

The pre-publication history for this paper can be accessed here:

http://www.biomedcentral.com/1471-2407/14/373/prepub

## References

[B1] Tinari N De Tursi M Grassadonia A Zilli M Stuppia L Iacobelli S Natoli C An epigenetic approach to pancreatic cancer treatment: the prospective role of histone deacetylase inhibitors Curr Cancer Drug Targets 2012 12 439 452 10.2174/156800912800190884 22309455

[B2] Siegel R DeSantis C Virgo K Stein K Mariotto A Smith T Cooper D Gansler T Lerro C Fedewa S Lin C Leach C Cannady RS Cho H Scoppa S Hachey M Kirch R Jemal A Ward E Cancer treatment and survivorship statistics, 2012 CA Cancer J Clin 2012 62 220 241 10.3322/caac.21149 22700443

[B3] Yeo CJ Cameron JL Improving results of pancreaticoduodenectomy for pancreatic cancer World J Surg 1999 23 907 912 10.1007/s002689900598 10449819

[B4] Assifi MM Hines OJ Anti-angiogenic agents in pancreatic cancer: a review Anticancer Agents Med Chem 2011 11 464 469 10.2174/187152011795677463 21521158

[B5] Andersson R Aho U Nilsson BI Peters GJ Pastor-Anglada M Rasch W Sandvold ML Gemcitabine chemoresistance in pancreatic cancer: molecular mechanisms and potential solutions Scand J Gastroenterol 2009 44 782 786 10.1080/00365520902745039 19214867

[B6] Weis SM Cheresh DA Tumor angiogenesis: molecular pathways and therapeutic targets Nat Med 2011 17 1359 1370 10.1038/nm.2537 22064426

[B7] Potente M Gerhardt H Carmeliet P Basic and therapeutic aspects of angiogenesis Cell 2011 146 873 887 10.1016/j.cell.2011.08.039 21925313

[B8] Folberg R Hendrix MJ Maniotis AJ Vasculogenic mimicry and tumor angiogenesis Am J Pathol 2000 156 361 381 10.1016/S0002-9440(10)64739-6 10666364 PMC1850026

[B9] Sun B Qie S Zhang S Sun T Zhao X Gao S Ni C Wang X Liu Y Zhang L Role and mechanism of vasculogenic mimicry in gastrointestinal stromal tumors Hum Pathol 2008 39 444 451 10.1016/j.humpath.2007.07.018 18261629

[B10] Guzman G Cotler SJ Lin AY Maniotis AJ Folberg R A pilot study of vasculogenic mimicry immunohistochemical expression in hepatocellular carcinoma Arch Pathol Lab Med 2007 131 1776 1781 18081435 10.5858/2007-131-1776-apsovm PMC2617786

[B11] Seftor RE Hess AR Seftor EA Kirschmann DA Hardy KM Margaryan NV Hendrix MJ Tumor cell vasculogenic mimicry: from controversy to therapeutic promise Am J Pathol 2012 181 1115 1125 10.1016/j.ajpath.2012.07.013 22944600 PMC4851740

[B12] Favier J Plouin PF Corvol P Gasc JM Angiogenesis and vascular architecture in pheochromocytomas: distinctive traits in malignant tumors Am J Pathol 2002 161 1235 1246 10.1016/S0002-9440(10)64400-8 12368197 PMC1867278

[B13] Lin SZ Wei WT Chen H Chen KJ Tong HF Wang ZH Ni ZL Liu HB Guo HC Liu DL Antitumor activity of emodin against pancreatic cancer depends on its dual role: promotion of apoptosis and suppression of angiogenesis PLoS One 2012 7 e42146 10.1371/journal.pone.0042146 22876305 PMC3410916

[B14] Qian DZ Kato Y Shabbeer S Wei Y Verheul HM Salumbides B Sanni T Atadja P Pili R Targeting tumor angiogenesis with histone deacetylase inhibitors: the hydroxamic acid derivative LBH589 Clin Cancer Res 2006 12 634 642 10.1158/1078-0432.CCR-05-1132 16428510

[B15] Kelly WK O’Connor OA Marks PA Histone deacetylase inhibitors: from target to clinical trials Expert Opin Investig Drugs 2002 11 1695 1713 10.1517/13543784.11.12.1695 12457432

[B16] Marks PA Discovery and development of SAHA as an anticancer agent Oncogene 2007 26 1351 1356 10.1038/sj.onc.1210204 17322921

[B17] Kumagai T Wakimoto N Yin D Gery S Kawamata N Takai N Komatsu N Chumakov A Imai Y Koeffler HP Histone deacetylase inhibitor, suberoylanilide hydroxamic acid (Vorinostat, SAHA) profoundly inhibits the growth of human pancreatic cancer cells Int J Cancer 2007 121 656 665 10.1002/ijc.22558 17417771

[B18] Ma X Yang Y Wang Y An G Lv G Small interfering RNA-directed knockdown of S100A4 decreases proliferation and invasiveness of osteosarcoma cells Cancer Lett 2010 299 171 181 10.1016/j.canlet.2010.08.016 20855150

[B19] Zhou Q Kiosses WB Liu J Schimmel P Tumor endothelial cell tube formation model for determining anti-angiogenic activity of a tRNA synthetase cytokine Methods 2008 44 190 195 10.1016/j.ymeth.2007.10.004 18241800 PMC3835186

[B20] Zhou Q Kapoor M Guo M Belani R Xu X Kiosses WB Hanan M Park C Armour E Do MH Nangle LA Schimmel P Yang XL Orthogonal use of a human tRNA synthetase active site to achieve multifunctionality Nat Struct Mol Biol 2010 17 57 61 10.1038/nsmb.1706 20010843 PMC3042952

[B21] Cao C Huang X Han Y Wan Y Birnbaumer L Feng GS Marshall J Jiang M Chu WM Galpha(i1) and Galpha(i3) are required for epidermal growth factor-mediated activation of the Akt-mTORC1 pathway Sci Signal 2009 2 ra17 19401591 10.1126/scisignal.2000118 PMC4138699

[B22] Cao C Sun Y Healey S Bi Z Hu G Wan S Kouttab N Chu W Wan Y EGFR-mediated expression of aquaporin-3 is involved in human skin fibroblast migration Biochem J 2006 400 225 234 10.1042/BJ20060816 16848764 PMC1652825

[B23] Roy SK Srivastava RK Shankar S Inhibition of PI3K/AKT and MAPK/ERK pathways causes activation of FOXO transcription factor, leading to cell cycle arrest and apoptosis in pancreatic cancer J Mol Signal 2010 5 10 10.1186/1750-2187-5-10 20642839 PMC2915986

[B24] Parsons CM Muilenburg D Bowles TL Virudachalam S Bold RJ The role of Akt activation in the response to chemotherapy in pancreatic cancer Anticancer Res 2010 30 3279 3289 20944098 PMC4557882

[B25] Furukawa T Molecular targeting therapy for pancreatic cancer: current knowledge and perspectives from bench to bedside J Gastroenterol 2008 43 905 911 10.1007/s00535-008-2226-1 19107333

[B26] Falasca M Selvaggi F Buus R Sulpizio S Edling CE Targeting phosphoinositide 3-kinase pathways in pancreatic cancer–from molecular signalling to clinical trials Anticancer Agents Med Chem 2011 11 455 463 10.2174/187152011795677382 21521159

[B27] Azzariti A Porcelli L Gatti G Nicolin A Paradiso A Synergic antiproliferative and antiangiogenic effects of EGFR and mTor inhibitors on pancreatic cancer cells Biochem Pharmacol 2008 75 1035 1044 10.1016/j.bcp.2007.11.018 18191814

[B28] He L Wu Y Lin L Wang J Chen Y Yi Z Liu M Pang X Hispidulin, a small flavonoid molecule, suppresses the angiogenesis and growth of human pancreatic cancer by targeting vascular endothelial growth factor receptor 2-mediated PI3K/Akt/mTOR signaling pathway Cancer Sci 2011 102 219 225 10.1111/j.1349-7006.2010.01778.x 21087351

[B29] Garrido-Laguna I Tan AC Uson M Angenendt M Ma WW Villaroel MC Zhao M Rajeshkumar NV Jimeno A Donehower R Iacobuzio-Donahue C Barrett M Rudek MA Rubio-Viqueira B Laheru D Hidalgo M Integrated preclinical and clinical development of mTOR inhibitors in pancreatic cancer Br J Cancer 2010 103 649 655 10.1038/sj.bjc.6605819 20664591 PMC2938261

[B30] Hess AR Seftor EA Seftor RE Hendrix MJ Phosphoinositide 3-kinase regulates membrane Type 1-matrix metalloproteinase (MMP) and MMP-2 activity during melanoma cell vasculogenic mimicry Cancer Res 2003 63 4757 4762 12941789

[B31] Mei J Gao Y Zhang L Cai X Qian Z Huang H Huang W VEGF-siRNA silencing induces apoptosis, inhibits proliferation and suppresses vasculogenic mimicry in osteosarcoma in vitro Exp Oncol 2008 30 29 34 18438338

[B32] Lu JH Shi ZF Xu H The mitochondrial cyclophilin D/p53 complexation mediates doxorubicin-induced non-apoptotic death of A549 lung cancer cells Mol Cell Biochem 2014 389 17 24 10.1007/s11010-013-1922-1 24343341

[B33] Glantschnig H Rodan GA Reszka AA Mapping of MST1 kinase sites of phosphorylation. Activation and autophosphorylation J Biol Chem 2002 277 42987 42996 10.1074/jbc.M208538200 12223493

[B34] Chen B Xu M Zhang H Wang JX Zheng P Gong L Wu GJ Dai T Cisplatin-induced non-apoptotic death of pancreatic cancer cells requires mitochondrial cyclophilin-D-p53 signaling Biochem Biophys Res Commun 2013 437 526 531 10.1016/j.bbrc.2013.06.103 23845906

[B35] Chen MB Wu XY Tao GQ Liu CY Chen J Wang LQ Lu PH Perifosine sensitizes curcumin-induced anti-colorectal cancer effects by targeting multiple signaling pathways both in vivo and in vitro Int J Cancer 2012 131 2487 2498 10.1002/ijc.27548 22438101

[B36] Hu M Xia M Chen X Lin Z Xu Y Ma Y Su L MicroRNA-141 regulates Smad interacting protein 1 (SIP1) and inhibits migration and invasion of colorectal cancer cells Dig Dis Sci 2010 55 2365 2372 10.1007/s10620-009-1008-9 19830559

[B37] Fabre M Garcia De Herreros A Phorbol ester-induced scattering of HT-29 human intestinal cancer cells is associated with down-modulation of E-cadherin J Cell Sci 1993 106 Pt 2 513 521 8282758 10.1242/jcs.106.2.513

[B38] Spalletta G Cravello L Piras F Iorio M Sancesario G Marchi A Caltagirone C Cacciari C Rapid-onset apathy may be the only clinical manifestation after dorsal striatum hemorrhagic lesion: a case report Alzheimer Dis Assoc Disord 2013 27 192 194 10.1097/WAD.0b013e318260ab97 22760169

[B39] Costello E Neoptolemos JP Pancreatic cancer in 2010: new insights for early intervention and detection Nat Rev Gastroenterol Hepatol 2011 8 71 73 10.1038/nrgastro.2010.214 21293504

[B40] Kim J Kim YC Fang C Russell RC Kim JH Fan W Liu R Zhong Q Guan KL Differential regulation of distinct Vps34 complexes by AMPK in nutrient stress and autophagy Cell 2013 152 290 303 10.1016/j.cell.2012.12.016 23332761 PMC3587159

[B41] Hideshima T Catley L Yasui H Ishitsuka K Raje N Mitsiades C Podar K Munshi NC Chauhan D Richardson PG Anderson KC Perifosine, an oral bioactive novel alkylphospholipid, inhibits Akt and induces in vitro and in vivo cytotoxicity in human multiple myeloma cells Blood 2006 107 4053 4062 10.1182/blood-2005-08-3434 16418332 PMC1895278

[B42] Vivanco I Sawyers CL The phosphatidylinositol 3-Kinase AKT pathway in human cancer Nat Rev Cancer 2002 2 489 501 10.1038/nrc839 12094235

[B43] Ravindran J Prasad S Aggarwal BB Curcumin and cancer cells: how many ways can curry kill tumor cells selectively? AAPS J 2009 11 495 510 10.1208/s12248-009-9128-x 19590964 PMC2758121

[B44] Duvoix A Blasius R Delhalle S Schnekenburger M Morceau F Henry E Dicato M Diederich M Chemopreventive and therapeutic effects of curcumin Cancer Lett 2005 223 181 190 10.1016/j.canlet.2004.09.041 15896452

[B45] Liu J Mao W Ding B Liang CS ERKs/p53 signal transduction pathway is involved in doxorubicin-induced apoptosis in H9c2 cells and cardiomyocytes Am J Physiol Heart Circ Physiol 2008 295 H1956 1965 10.1152/ajpheart.00407.2008 18775851 PMC2614569

