# Erratum to: Suberoylanilide hydroxamic acid, an inhibitor of histone deacetylase, suppresses vasculogenic mimicry and proliferation of highly aggressive pancreatic cancer PaTu8988 cells

**DOI:** 10.1186/s12885-016-2401-3

**Published:** 2016-07-04

**Authors:** Xing-dong Xu, Lan Yang, Li-yun Zheng, Yan-yan Pan, Zhi-fei Cao, Zhi-qing Zhang, Quan-sheng Zhou, Bo Yang, Cong Cao

**Affiliations:** Department of General Surgery, the Third Hospital affiliated to Soochow University, Changzhou City, Jiangsu 213003 China; Department of Neurology, the First Affiliated Hospital of Soochow University, Suzhou, China; Cyrus Tang Hematology Center, Soochow University, Suzhou, Jiangsu 215123 China; Jiangsu Key Laboratory of Translational Research and Therapy for Neuro-Psycho-Diseases and Institute of Neuroscience, Soochow University, Suzhou, Jiangsu 215021 China

## Erratum

Unfortunately, the original version of this article [1] contained an error within Fig. 5. For testing Sema4D protein expression in PaTu8988 cells with/out SAHA treatment, the protein samples of Fig. 2D were re-tested by Western blot assay using Sema4D antibody (Fig. 5E). The correct version of Fig. 5E can be found below. This does not alter the conclusion of the result.

**Fig. 5:**
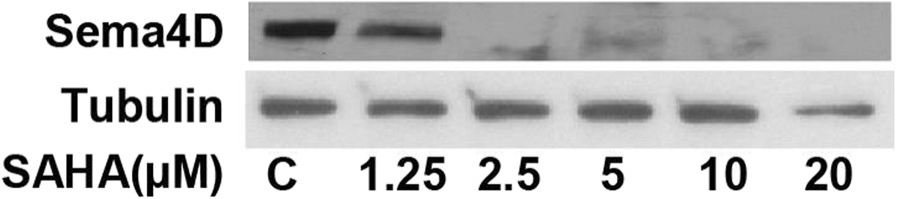
PaTu8988 cells were incubated with SAHA at indicated dosage for 48 hours, the protein expressions of Sema4D and tubulin were tested by Western blots. Experiments in this figure were repeated three times, and similar results were obtained. **e** SAHA suppresses PaTu8988 cell vasculogenic mimicry (VM)
